# Bayesian federated inference for survival models

**DOI:** 10.1080/02664763.2025.2511932

**Published:** 2025-06-04

**Authors:** Hassan Pazira, Emanuele Massa, Jetty A.M. Weijers, Anthony C.C. Coolen, Marianne A. Jonker

**Affiliations:** a Research Institute for Medical Innovation, Science department IQ Health, Research & Education group Biostatistics, Radboud University Medical Center, Nijmegen, Netherlands; b Donders Institute, Faculty of Science, Radboud University, Nijmegen, Netherlands; c Radboud Institute for Medical Innovation, Department of Medical Oncology, Radboud University Medical Center, Nijmegen, Netherlands; d Saddle Point Science Europe, Mercator Science Park, Nijmegen, Netherlands

**Keywords:** Decentralized data, distributed inference, federated learning, one-shot algorithm, rare cancer, 91G70, 62F07, 62P10, 62N02, 62F15

## Abstract

To accurately estimate the parameters in a prediction model for survival data, sufficient events need to be observed compared to the number of model parameters. In practice, this is often a problem. Merging data sets from different medical centers may help, but this is not always possible due to strict privacy legislation and logistic difficulties. Recently, the Bayesian Federated Inference (BFI) strategy for generalized linear models was proposed. With this strategy, the statistical analyzes are performed in the local centers where the data were collected (or stored), and only the inference results are combined to a single estimated model; merging data is not necessary. The BFI methodology aims to compute from the separate inference results in the local centers what would have been obtained if the analysis had been based on the merged data sets. In the present paper, we generalize the BFI methodology as initially developed for generalized linear models to survival models. Simulation studies and real data analyzes show excellent performance; that is, the results obtained with the BFI methodology are very similar to the results obtained by analyzing the merged data. An R package for doing the analyzes is available.

## 1. Introduction

In cancer research, but also in many other research fields, overall survival is frequently regarded as the outcome of main interest; it is seen as the gold standard. To ensure sufficient statistical power to infer the efficacy of a treatment on overall survival or to accurately estimate an overall survival prediction model, a sufficient number of events (i.e. deaths) must be present in the data set. The latter may be a problem, especially if survival rates are reasonably high. This has led to a shift from overall survival as the primary outcome to surrogate outcomes, such as progression-free survival and the objective response rate. An alternative approach would be to enlarge the dataset and increase the total number of events (deaths) by sharing data across different medical centers. Because of regulatory constraints and privacy legislation, this is not always possible, especially if the medical centers are located in different countries.

Recently, in [5] the so-called Bayesian Federated Inference (BFI) methodology for generalized linear models (GLMs) was proposed, to extract and combine information from different medical centers without actually sharing the data. In each center the local data are analyzed and the inference results are sent to a central server, where the results from the separate medical centers are combined into one statistical model such that the estimated parameters are approximately equal to the estimates which would have been obtained if the data sets were pooled before the analysis was performed. An expression of the BFI estimator of the model parameters in terms of the local estimators is found in steps. First, the log posterior density for the fictive combined data set is written in terms of the local log posterior densities. Second, these local log densities are approximated by a second-order Taylor expansion around their local maximum a prosteriori (MAP) estimators. The BFI estimator is defined as the value that maximizes the obtained approximation of the log posterior density for the combined data set. With this methodology, privacy issues do not play any role as the patient data do not leave the hospital and can not be retrieved from the inference results that are sent to the central server.

The BFI methodology for GLMs is valid under conditions of homogeneity and certain types of heterogeneity across centers, with estimators being asymptotically efficient in both settings [5,6]. Explicit expressions of the BFI estimators in terms of the local center estimators have been derived. Once the local estimates are available, the BFI estimates can be computed using these expressions. If analyzes in some centers are delayed or new centers are added in the study, the BFI estimates can be updated relatively easily without requiring additional contact with the centers. In a medical setting it is important that the local analyzes have to performed once only, since support from the medical and possibly statistical staff is needed for this.

Methodology for federated analyzes is not new. Federated Learning (FL) is an iterative machine learning approach that was developed several years ago [10]. Its aim is the same as that of the BFI method: doing a combined analysis without sharing the data. However, the methods differ in implementation. In FL only the local data are used to train a machine learning system in each center. The locally estimated parameters are sent to the central server where the model is updated. The updated model is sent to the local centers again where the parameters are re-estimated based on the local data and the updated model. This process is iterated across centers until convergence is reached, often requiring over 100 iterations. As a comparison, for the BFI methodology a single estimate, so a single round, is sufficient. If the local centers are hospitals (the setting we are interested in), a cycling mechanism is complex and often not convenient for medical doctors; a one-shot approach like the BFI is preferred. The FL strategy has evolved enormously in recent years [9]. It performs excellently in e.g. image analysis, [2,12], but cannot easily handle complex statistical models, which are often encountered in medical data analysis.

In the present paper we generalize the BFI methodology developed for GLMs to survival models for prediction. The Cox PH model is a semi-parametric model for analyzing survival data. Its hazard function equals a product of a non-parametric baseline hazard function and a parametric regression part that depends on the covariates. The regression parameters are estimated by maximizing the partial likelihood which does not depend on the baseline hazard function. For the situation that data from different centers can not be combined, single and multiple communication strategies have been proposed to estimate the regression parameters [4,8,15]. None of these methods, however, estimate the baseline hazard function. For predicting the survival time of future patients, estimates of both the regression parameters and the baseline hazard function are needed. Therefore, methods that do not estimate the baseline hazard function are not useful for our research setting. In the present paper, the BFI methodology is developed for parametric survival models and BFI estimators are derived for all parameters, including those of the baseline hazard function. Specifically, we consider several parametric baseline hazard functions of a fixed form (exponential, Weibull, Gompertz), an exponentiated polynomial hazard function, and the piecewise constant hazard function. Asymptotically, the BFI estimators are efficient. Their finite sample behavior is studied by means of simulations and the results are compared with those of the weighted average estimator (computed as the average of the local estimates weighted by their local sample sizes). Furthermore, it is explained how the methodology can be generalized to deal with some forms of heterogeneity between centers. One of the motivations for our research was the estimation of overall survival models for salivary gland cancer, a rare cancer. As an application we analyze data of patients with this cancer type.

The outline of the paper is as follows. In Section 2 the methodology of the BFI for a general survival models is derived for a parametric baseline hazard function. Different choices for this baseline hazard function are described in Section 3. Next, in Section 4 the results of multiple simulation studies are presented to study the performance of the BFI methodology for the different baseline hazard functions. A BFI analysis of data of salivary gland cancer patients is described in Section 5. The paper is summarized and discussed in Section 6. In that section we also discuss some points for improvement which will be addressed in later projects. The BFI methodology for GLMs and survival models has been implemented in an R-package called BFI and an accompanying manual is available [11].

## 2. Bayesian federated inference for survival models

In this section we explain how to extend the Bayesian Federated Inference methodology as was originally proposed in [5], to models for time to event data. We consider the scenario in which data in *L* medical centers are available, but these data can not be merged to a single data set.

### 2.1. Setting and notation

Let, for patient *i* from center ℓ, $Y_{\mathrm{\ell i}}$ be the time from a well specified time zero to an event of interest. The patient's vector of covariates measured at time zero is denoted as $Z_{\mathrm{\ell i}}\in R^{p}$. Suppose that $Y_{\mathrm{\ell i}}|(Z_{\mathrm{\ell i}}=z,\theta )$ has density y∣z,θ↦p(y∣z,θ) and survival function $y|z,\theta \mapsto S(y|z,\theta )=\int_{y}^{\infty }p(s|z,\theta )\;ds$, that are known up to the parameter θ which itself has a density function $\theta \mapsto \pi_{\ell }(\theta )$. Let $C_{\mathrm{\ell i}}$ be a non-negative right censoring time for this patient. We assume that conditional on $Z_{\mathrm{\ell i}}$ (and the parameters), the censoring time $C_{\mathrm{\ell i}}$ is independent of $Y_{\mathrm{\ell i}}$. Further, let $T_{\mathrm{\ell i}}$ be defined as $T_{\mathrm{\ell i}}=\min\{Y_{\mathrm{\ell i}},C_{\mathrm{\ell i}}\}$ and let $\Delta_{\mathrm{\ell i}}=1\{T_{\mathrm{\ell i}}\leq C_{\mathrm{\ell i}}\}$ be the indicator that equals 1 if $T_{\mathrm{\ell i}}\leq C_{\mathrm{\ell i}}$ and 0 otherwise. There are *L* data sets in *L* centers which can not be merged. We assume that the patients' (stochastic) variables within the centers and across the centers are independent and identically distributed (conditional on the model parameters).

For patient *i* from center ℓ we observe the realization $\left(t_{\mathrm{\ell i}},\delta_{\mathrm{\ell i}},z_{\mathrm{\ell i}}\right)$ of the stochastic triple $\left(T_{\mathrm{\ell i}},\Delta_{\mathrm{\ell i}},Z_{\mathrm{\ell i}}\right)$. The data set in center ℓ is denoted by $D_{\ell }$:

$$D_{\ell }=\left\{\left(t_{\ell 1},\delta_{\ell 1},z_{\ell 1}\right),\ldots ,\left(t_{\ell n_{\ell }},\delta_{\ell n_{\ell }},z_{\ell n_{\ell }}\right)\right\},$$

where $n_{\ell }=|D_{\ell }|$ is the number of patients in data set $D_{\ell }$. The (fictive) combined data set, which is never actually created, is denoted as $D=\cup_{\ell =1}^{L}D_{\ell }$ and contains data of $n:=|D|=\sum_{\ell =1}^{L}n_{\ell }$ patients.

Different survival models for the time to event variable $Y_{\mathrm{\ell i}}$ correspond to different functional forms for p(y∣z,θ). Conditional on the vector of covariates $Z_{\mathrm{\ell i}}=z$ and θ, the hazard function for $Y_{\mathrm{\ell i}}$ equals

$$y|z,\theta \mapsto \lambda (y|z,\theta )=\lambda_{0}(y|\omega )\exp(z^{⊤}\beta ),\;\theta =(\beta ,\omega {)}^{⊤}$$

where $\lambda_{0}(\cdot |\omega )$ is the baseline hazard function which is known up to the parameter $\omega \in R^{q}$, and $\beta \in R^{p}$ is the unknown vector with regression parameters. This yields the conditional density of the form

$$y|z,\theta \mapsto p(y|z,\theta )=\lambda_{0}(y|\omega )\exp\left\{z^{⊤}\beta -\Lambda_{0}(y|\omega )\exp(z^{⊤}\beta )\right\},$$

where $\Lambda_{0}(y|\omega )=\int_{0}^{y}\lambda_{0}(s|\omega )\;ds$ is the cumulative baseline hazard function at time *y*. This class of models gives, for data set $D_{\ell }$, the likelihood function

$$p(D_{\ell }|\beta ,\omega )\propto \prod_{i=1}^{n_{\ell }}{\left\{\lambda_{0}(t_{\mathrm{\ell i}}|\omega )\exp(z_{\mathrm{\ell i}}^{⊤}\beta )\right\}}^{\delta_{\mathrm{\ell i}}}\exp\left\{-\Lambda_{0}(t_{\mathrm{\ell i}}|\omega )\exp(z_{\mathrm{\ell i}}^{⊤}\beta )\right\},$$

where we left out the factors that depend on the distribution for the censoring times and the covariates only and, thus, do not depend on the parameters of interest $\theta =(\beta ,\omega {)}^{⊤}$.

Suppose it had been possible to combine the data into the large data set D. For D, let (β,ω)↦π(β,ω) be the *global* prior over the parameters of the model. Then, its posterior density (β,ω)↦p(β,ω∣D) would have been given by

$$p(\beta ,\omega |D)=\frac{p(D|\beta ,\omega )\pi (\beta ,\omega )}{Z(D)}=\frac{\exp\left\{\Omega (\beta ,\omega |D)\right\}}{Z(D)},$$

with Z(D):=∫p(D∣β,ω)π(β,ω) dβ dω the normalizing constant of the posterior distribution and

(1) Ω(β,ω∣D)=log⁡{π(β,ω)}+log⁡{p(D∣β,ω)}.

The maximum a posteriori (MAP) estimator for (β,ω), denoted as $\left(\hat{\beta },\hat{\omega }\right)$, is obtained by maximizing (β,ω)↦p(β,ω∣D) with respect to (β,ω):

$$(\hat{\beta },\hat{\omega }):=\underset{\left(\beta ,\omega \right)}{\arg\max}\;p(\beta ,\omega |D)=\underset{\left(\beta ,\omega \right)}{\arg\max}\;\Omega (\beta ,\omega |D).$$

Data sharing, i.e. creating D, is often not possible, hence the above *global* MAP estimator cannot be computed. Bayesian Federated Inference seeks to obviate this issue by deriving an estimator for (β,ω) which is an expression of the *local* MAP estimators, i.e. the modes of the *local* posteriors $\left(\beta ,\omega )\mapsto p_{\ell }(\beta ,\omega |D_{\ell }\right)$:

(2) $$\left({\hat{\beta }}_{\ell },{\hat{\omega }}_{\ell }\right):=\underset{\left(\beta ,\omega \right)}{\arg\max}\;p_{\ell }\left(\beta ,\omega |D_{\ell }\right)=\underset{\left(\beta ,\omega \right)}{\arg\max}\;\Omega_{\ell }(\beta ,\omega |D_{\ell })$$

with

$$\Omega_{\ell }\left(\beta ,\omega |D_{\ell }\right)=\log\left\{\pi_{\ell }(\beta ,\omega )\right\}+\log\left\{p(D_{\ell }|\beta ,\omega )\right\}$$

where $\left(\beta ,\omega )\mapsto \pi_{\ell }(\beta ,\omega \right)$ is the *local* prior for the model parameters, and

$$\log\left\{p(D_{\ell }|\beta ,\omega )\right\}=\sum_{i=1}^{n_{\ell }}\left\{\delta_{\mathrm{\ell i}}\left(z_{\mathrm{\ell i}}^{⊤}\beta +\log\{\lambda_{0}(t_{\mathrm{\ell i}}|\omega )\}\right)-\Lambda_{0}(t_{\mathrm{\ell i}}|\omega )\exp(z_{\mathrm{\ell i}}^{⊤}\beta )\right\}.$$

In the next subsection the BFI estimator is derived. This estimator is an expression of the local MAP estimators $({\hat{\beta }}_{\ell },{\hat{\omega }}_{\ell }),\ell =1,\ldots ,L$. Then, once the local MAP estimates have been computed in the centers and sent to the central server, (β,ω) can be estimated via this expression.

### 2.2. Deriving the BFI estimators

By the definition of Ω(β,ω∣D) in (1) and the statistical independence between the data across the different centers, Ω(β,ω∣D) can be written as

(3) $$\begin{array}{rl} \Omega (\beta ,\omega |D) & \;=\log\{\pi (\beta ,\omega )\}+\log\{p(D|\beta ,\omega )\}=\log\{\pi (\beta ,\omega )\}+\sum_{\ell =1}^{L}\log\{p(D_{\ell }|\beta ,\omega )\}\\ & \;=\sum_{\ell =1}^{L}\Omega_{\ell }(\beta ,\omega |D_{\ell })+\log\{\pi (\beta ,\omega )\}-\sum_{\ell =1}^{L}\log\{\pi_{\ell }(\beta ,\omega )\}. \end{array}$$

We approximate the local posteriors by a second order Taylor expansion around the local MAP estimators $\left({\hat{\beta }}_{\ell },{\hat{\omega }}_{\ell }\right)$:

$$\begin{array}{rl} \Omega_{\ell }(\beta ,\omega |D_{\ell }) & \;=\Omega_{\ell }({\hat{\beta }}_{\ell },{\hat{\omega }}_{\ell }|D_{\ell })-\frac{1}{2}{\left(\begin{array}{c} \beta -{\hat{\beta }}_{\ell }\\ \omega -{\hat{\omega }}_{\ell } \end{array}\right)}^{⊤}{\hat{M}}_{\ell }\left(\begin{array}{c} \beta -{\hat{\beta }}_{\ell }\\ \omega -{\hat{\omega }}_{\ell } \end{array}\right)\\ & \;\;+O_{p}\left(\|\beta -{\hat{\beta }}_{\ell }{\|}^{3}+\|\omega -{\hat{\omega }}_{\ell }{\|}^{3}\right) \end{array}$$

where $O_{p}(\|\beta -{\hat{\beta }}_{\ell }{\|}^{3}+\|\omega -{\hat{\omega }}_{\ell }{\|}^{3})$ is the remainder term that is of the order $\|\beta -{\hat{\beta }}_{\ell }{\|}^{3}+\|\omega -{\hat{\omega }}_{\ell }{\|}^{3}$ for the sample size going to infinity [13] and where the matrix ${\hat{M}}_{\ell }$ is equal to minus the Hessian matrix of $\left(\beta ,\omega )\mapsto \Omega_{\ell }(\beta ,\omega |D_{\ell }\right)$ evaluated at the MAP estimator. The linear term is missing in the expansion as this term equals zero by the definition of the MAP estimator. Furthermore, we assume zero mean Gaussian priors in the combined and local data sets, with inverse covariance matrices Γ and $\Gamma_{\ell }$. Inserting the Taylor approximation of $\Omega_{\ell }(\beta ,\omega |D_{\ell })$ and the Gaussian prior densities into the expression of Ω(β,ω∣D) in (3), gives

(4) $$\Omega (\beta ,\omega |D)=\Omega_{\text{\{BFI\}}}(\beta ,\omega )+O_{p}\left(\max_{\ell =1,\ldots ,L}\left\{\|\beta -{\hat{\beta }}_{\ell }{\|}^{3}+\|\omega -{\hat{\omega }}_{\ell }{\|}^{3}\right\}\right)$$

where the BFI surrogate objective function $\left(\beta ,\omega )\mapsto \Omega_{\text{\{BFI\}}}(\beta ,\omega \right)$ is defined as

$$\begin{array}{rl} \Omega_{\text{\{BFI\}}}(\beta ,\omega ) & \;:=\sum_{\ell =1}^{L}\left\{\Omega_{\ell }({\hat{\beta }}_{\ell },{\hat{\omega }}_{\ell }|D_{\ell })-\frac{1}{2}{\left(\begin{array}{c} \beta -{\hat{\beta }}_{\ell }\\ \omega -{\hat{\omega }}_{\ell } \end{array}\right)}^{⊤}{\hat{M}}_{\ell }\left(\begin{array}{c} \beta -{\hat{\beta }}_{\ell }\\ \omega -{\hat{\omega }}_{\ell } \end{array}\right)\right\}\\ & \;\;-\frac{1}{2}{\left(\begin{array}{c} \beta \\ \omega \end{array}\right)}^{⊤}\left(\Gamma -\sum_{\ell =1}^{L}\Gamma_{\ell }\right)\left(\begin{array}{c} \beta \\ \omega \end{array}\right). \end{array}$$

The function $\left(\beta ,\omega )\mapsto \Omega_{\text{\{BFI\}}}(\beta ,\omega \right)$ is a quadratic function with respect to β and ω and can be easily maximized with respect to (β,ω). The value in which $\Omega_{\text{\{BFI\}}}(\beta ,\omega )$ attains its maximum is denoted as $\left({\hat{\beta }}_{\text{\{BFI\}}},{\hat{\omega }}_{\text{\{BFI\}}}\right)$, and is given by

(5) $$\begin{array}{r} \left(\begin{array}{c} {\hat{\beta }}_{\text{\{BFI\}}}\\ {\hat{\omega }}_{\text{\{BFI\}}} \end{array}\right)={\hat{M}}_{\text{\{BFI\}}}^{-1}\sum_{\ell =1}^{L}{\hat{M}}_{\ell }\left(\begin{array}{c} {\hat{\beta }}_{\ell }\\ {\hat{\omega }}_{\ell } \end{array}\right),\;{\hat{M}}_{\text{\{BFI\}}}=\Gamma +\sum_{\ell =1}^{L}\left({\hat{M}}_{\ell }-\Gamma_{\ell }\right). \end{array}$$

For ${\hat{\beta }}_{\ell }$ and ${\hat{\omega }}_{\ell }$ in a small neighborhood of $\left({\hat{\beta }}_{\text{\{BFI\}}},{\hat{\omega }}_{\text{\{BFI\}}}\right)$ for every ℓ, the remainder term in (4) will be small compared to $\Omega_{\text{\{BFI\}}}({\hat{\beta }}_{\text{\{BFI\}}},{\hat{\omega }}_{\text{\{BFI\}}})$. If, moreover, this remainder term behaves well in the sense that it is of bounded variation, the estimator $\left({\hat{\beta }}_{\text{\{BFI\}}},{\hat{\omega }}_{\text{\{BFI\}}}\right)$ will be close to global MAP estimators in the combined data set: $\left(\hat{\beta },\hat{\omega }\right)$.

We stress that the BFI estimators can be computed via linear operations in a single communication round from the local centers to the global central server *without* sharing the data, but only the local MAP estimates $\left({\hat{\beta }}_{\ell },{\hat{\omega }}_{\ell },{\hat{M}}_{\ell }\right)$. An iterative algorithm to obtain estimators, as would have been required in Federated Learning procedures, is here not necessary.

In [6] it is proven that the BFI estimator for the parameters in GLMs is asymptotically efficient. The proof also holds for the parametric survival models considered in the present paper. It follows that the BFI estimator in (5) is asymptotically Gaussian with mean zero and a covariance matrix that can be estimated by the asymptotically consistent estimator ${\hat{M}}_{\text{\{BFI\}}}$. With this asymptotic result, the following expression for the approximated credible intervals for the parameters can be derived: for the $k^{\mathrm{th}}$ element of (β,ω) its approximate (1−2α)100% credible interval equals $({\hat{\beta }}_{\text{\{BFI\}}},{\hat{\omega }}_{\text{\{BFI\}}}{)}_{k}\pm \xi_{\alpha }\;({\hat{M}}_{\text{\{BFI\}}}^{-1}{)}_{k,k}^{1/2},$ for $\xi_{\alpha }$ the upper *α*-quantile of the standard Gaussian distribution.

In the present section, the expression of the BFI estimator is derived under the assumption of homogeneity across the centers. That means that it is assumed that the value of the parameter vector (β,ω) is the same across the centers. This is a strong assumption and may be violated if the populations in the centers differ. Heterogeneity due to differences in the distributions of the covariates (and not the parameters of the regression model conditional the covariates) does not effect the BFI estimator of (β,ω). If survival in some centers is clearly lower than in other centers and this cannot be explained by the covariates in the model, there may be heterogeneity in the parameters of the baseline hazard function. This can be taken into account with the BFI methodology. The MAP estimates of the parameters that are assumed to be the same across centers are still combined into a single BFI estimate, while the estimates of the parameters that may differ across centers are modeled center-specific. In [6] new expressions of the BFI estimators have been derived for the parameters in GLMs under different types of heterogeneity. These derivations can be extended easily to the survival models. In Section 3 heterogeneity is discussed briefly for every choice of the baseline hazard function.

## 3. Choices for the baseline hazard function

In the previous section we derived the BFI estimators for a parametric survival model in which the baseline hazard function has a general parametric form. In this section we consider different choices for this form. In Section 3.1 we start with the Weibull and Gompertz parameterizations of the baseline hazard, which depend on only two parameters. We then continue with more complex models, where the number of parameters can, in principle, be increased arbitrarily, allowing for more flexibility (i.e. as the number of parameters increases, the model can more accurately represent the shape of the underlying true function). In particular in Section 3.2 we consider the simple, yet quite flexible, Piece-Wise Exponential model, where, as the name suggests, the baseline hazard function is approximated with a piece-wise constant function. Finally, Section 3.3 deals with the Exponentiated Polynomial parameterization, where the logarithm of the baseline hazard function is assumed to be a polynomial.

If categorical variables are included in the model, one of the categories is seen as reference category, and for the other categories a dummy-variable is defined. In most regression models the effect of the reference group is hidden in the intercept. In the semi-parametric Cox model no intercept is included as this intercept is incorporated in the baseline hazard function. If the baseline hazard function is parametric, the intercept need to be included in one of the parameters of the baseline hazard function. From the functional form of the baseline hazard functions given below, it is clear that this happens.

### 3.1. Simple parametric proportional hazards models

The models discussed in this subsection are parameterized by a scale parameter $\exp(\omega_{1})\in R^{+}$ where $\omega_{1}\in R$ and a location parameter $\exp(\omega_{2})\in R^{+}$ with $\omega_{2}\in R$. Specifically, we examine the Weibull and Gompertz parameterizations of the baseline hazard rate. These are well-known simple parametric models that assume fixed functional forms for the baseline hazard function.

In the Weibull model the baseline hazard function and the cumulative baseline hazard functions are defined by

$$\lambda_{0}(t|\omega_{1},\omega_{2})=\exp(\omega_{1}+\omega_{2})t^{\exp(\omega_{2})-1}\;\mathrm{and}\;\Lambda_{0}(t|\omega_{1},\omega_{2})=\exp(\omega_{1})t^{\exp(\omega_{2})}.$$

To compute the matrix ${\hat{M}}_{\ell }$ the first and second derivatives of the cumulative baseline hazard function with respect to the parameters must be calculated.

In the Gompertz model the baseline hazard function and the cumulative baseline hazard function are equal to

$$\lambda_{0}(t|\omega_{1},\omega_{2})=\exp(\omega_{1}+\exp(\omega_{2})t),$$

and

$$\Lambda_{0}(t|\omega_{1},\omega_{2})=\exp(\omega_{1}-\omega_{2})\left(\exp(\exp(\omega_{2})t)-1\right).$$

Both Weibull and Gompertz parameterizations of the baseline hazard function $\lambda_{0}(\cdot )$ are monotonic: the Gompertz baseline hazard function is increasing, while the Weibull baseline hazard function might be increasing ( $\omega_{2}>1$) or decreasing ( $\omega_{2}<1$).

In the Weibull and Gompertz model, the parameter $\omega_{1}$ plays a role similar to the intercept of the regression model. Differences in survival between centers that cannot be explained by variation in the covariate distributions, can be taken into account by allowing center specific intercepts (i.e. a center specific parameter $\omega_{1}$). Expressions for the corresponding BFI estimators have been derived in [6]. Heterogeneity in $\omega_{2}$ can be accounted for in a similar way.

### 3.2. Piece-wise exponential model

In the piecewise exponential model, the baseline hazard function is approximated by a function which is constant on *q* intervals delimited by *q* + 1 end points $\tau_{0},\ldots ,\tau_{q}$. The basis functions are defined as

(6) $$b(t)={\left(b_{0}(t),\ldots ,b_{q-1}(t)\right)}^{⊤},\;b_{k}(t):=I[\tau_{k}<t<\tau_{k+1}],\;k=0,\ldots ,q-1,$$

where $I[\tau_{k}<t<\tau_{k+1}]$ equals 1 if $\tau_{k}<t<\tau_{k+1}$ and 0 otherwise. For these basis functions the baseline hazard function can be written as

$$\lambda_{0}(t|\omega )=\exp(\omega {)}^{⊤}b(t)$$

with $\exp(\omega )=(\exp(\omega_{0}),\ldots ,\exp(\omega_{q-1}){)}^{⊤}$ denoting the unknown parameters that weigh the different basis functions. These are the parameters that need to be estimated. This gives the cumulative hazard function $\Lambda_{0}(t|\omega )=\exp(\omega {)}^{⊤}B(t),$ where

$$B(t)={\left(B_{0}(t),\ldots ,B_{q-1}(t)\right)}^{⊤},\;B_{k}(t):=I[t>\tau_{k}]\min\{t-\tau_{k},\tau_{k+1}-\tau_{k}\}.$$

The piece-wise parametrization can be regarded as a relatively simple fully parametric version of the Cox model. Its advantage over the parametric models of the previous subsection is its flexibility. In fact the piece-wise exponential model is a special case of a B-spline parameterization of the baseline hazard function [7]. The choice of the knots (i.e. $\tau_{1},\ldots ,\tau_{q-1}$) plays a central role: ‘well chosen’ knots will lead to a better fit of the data. Although several recipes have been put forward, there is no unique agreed methodology to choose the position of the knots. It has been noticed in literature [3] that the positions of the knots do not generally impact on the quality of the fit: the number of knots is crucial. Within the BFI framework it is essential that the same knots are used in every center. Practically it might well be that the researchers across the different centers agree on a reasonable placement of the knots. Alternatively it is also possible to adopt a penalized splines (P-spline) approach, i.e. use a large number of equi-spaced knots and introduce a penalty term to contain the model complexity and ‘mitigate’ overfitting.

In the piece-wise exponential model, heterogeneity of the baseline hazard functions across the centers can be accounted for in the BFI framework by allowing different parameters ω across the centers. Only the parameters that are assumed to be homogeneous between the centers are combined with the BFI methodology.

### 3.3. Exponentiated polynomial model

In the exponentiated polynomial model, the baseline hazard function is equal to

$$\lambda_{0}(t|\omega )=\exp\left\{\omega^{⊤}b(t)\right\}$$

where ω is a *q*-dimensional vector of unknown parameters and $b(t)=(b_{0}(t),\ldots ,b_{q-1}(t){)}^{⊤}$ with $b_{k}(t)=t^{k},k=0,\ldots ,q-1$. So the function $\omega^{⊤}b(t)$ is equal to a polynomial of the order *q*−1. The cumulative baseline hazard function cannot be computed in a closed form:

$$\Lambda_{0}(t|\omega )=\int_{0}^{t}\exp\left\{\omega^{⊤}b(s)\right\}\;ds$$

which must then be evaluated numerically (e.g. when maximizing the posterior density), e.g. via quadratures.

In the BFI framework it is important that the models that are fitted in the local centers have the same polynomial order. Possibly, the researchers across the different local centers can agree upon the model, or the researcher in the central server can determine this. Since the models are nested, there is hardly any risk if the order is chosen slightly too high; the estimates of the parameters belonging to the redundant higher order terms will be close to zero. If the order is chosen too small, the estimated baseline hazard function will be less flexible than it should be.

It might be difficult to choose an appropriate order beforehand. The order for the polynomial function can also be chosen based on statistical arguments. The idea is that in each local center the model with the ‘best’ polynomial order is chosen based on a statistical procedure. For $q_{\ell }^{⋆}$ the chosen order in center ℓ, this yields the orders $q_{1}^{⋆},\ldots ,q_{L}^{⋆}$ in the *L* centers. Since our initial assumption is that the models in all centers share the same baseline hazard function, the order of the model is set equal to the maximum of the local orders $q^{⋆}=\max\{q_{1}^{⋆},\ldots ,q_{L}^{⋆}\}$. Next, in every center a model with order $q^{⋆}$ is fitted and the *L* estimated models are combined with the BFI methodology to obtain a single model for the merged data. The motivation for taking the highest-order polynomial is that it should capture more complex features and details in the combined data and thus estimate the baseline hazard function more accurately. Moreover, the models are nested and the model with a higher order includes the models with lower orders.

One way to select the optimal local order within a center is by using the likelihood ratio test. Let $M_{q}$ be the model with a polynomial of order *q*. Note that the models form a sequence of nested models; model $M_{0}$ is a special case of model $M_{1}$, model $M_{1}$ is embedded into $M_{2}$, and so on. In every center, first the null hypothesis $H_{0}:M_{0}=M_{1}$ is tested against the alternative hypothesis $H_{1}:M_{0}\neq M_{1}$. The model $M_{0}$ is the simplest model where the baseline hazard function is constant over time (i.e. the exponential model), and model $M_{1}$ assumes a baseline hazard function that is exponential in time. If $H_{0}$ is not rejected, then the procedure ends, and we choose model $M_{0}$ as the ‘best’ model given the data in the local center. Otherwise, the procedure continues with testing the null hypothesis $H_{0}:M_{1}=M_{2}$ against $H_{1}:M_{1}\neq M_{2}$, and so forth until the null hypothesis is not rejected.

In the exponentiated polynomial model heterogeneity in terms of varying intercepts, can again be included by allowing the first parameter of ω to vary across the centers.

## 4. Simulation studies

### 4.1. Settings

Suppose there are *L* = 3 centers with data sets with sample sizes varying between 50, 100 and 500. For each patient the time-to-event is simulated from a multivariable model with four covariates. The baseline hazard function correspond to the Weibull distribution with a scale parameter of $\exp(\omega_{1})$ where $\omega_{1}=-0.9$ and a shape parameter of $\exp(\omega_{2})$ with $\omega_{2}=1.8$. Within a patient (and across patients) the covariate values are independently and randomly generated from a standard normal distribution. The values of the regression coefficients are set equal to β=(−0.6,−0.4,0.4,0.6) to achieve hazard ratios ranging from about 0.5 to 2. For generating the survival data with a predefined censoring rate of 30%, we used a modified version of the methodology proposed by [14] (see the Appendix).

For analysis, we consider six models: exponential (Exp), Weibull (Wei), Gompertz (Gom), exponentiated polynomial (Poly), piece-wise exponential with four intervals (PW4) and piece-wise exponential with eight intervals (PW8). Further, we assumed zero-mean multivariate Gaussian distribution as (relatively uninformative) priors with the inverse covariance matrix equal to a diagonal matrix $\Gamma_{\beta }=\Gamma_{\beta \ell }=10^{-2}I_{4}$ and $\Gamma_{\omega }=\Gamma_{\omega \ell }=10^{-2}I_{q}$ where *q* refers to the number of model parameters for the baseline hazard function. For example, in the PW4 model *q* = 4. For the Poly model we set a maximum value for *q* to be 2 (i.e. $\omega_{0}$ and $\omega_{1}$), corresponding to a first order polynomial. For each choice of the sample sizes in the centers, data sets are simulated and analyzed *B* = 100 times.

For comparison, we consider two more estimators which are commonly used in practice if the data from different centers can not be merged. The first estimator is referred to as the weighted average estimator (WAV). This estimator is found by combining the MAP estimators from the *L* different data sets by taking a weighted average where the weights are based on the size of the data set. The estimator for the $k^{\mathrm{th}}$ coordinate is defined as:

$${\hat{\beta }}_{\text{\{WAV\}},k}=\sum_{\ell =1}^{L}\frac{n_{\ell }}{n}\;{\hat{\beta }}_{\ell ,k},$$

where $n=\sum_{\ell =1}^{L}n_{\ell }$. The second extra estimator is simply the MAP estimator in the largest of the *L* data sets. We refer to this estimator as ${\hat{\beta }}_{\text{\{single\}}}$ and to its $k^{\mathrm{th}}$ coordinate as ${\hat{\beta }}_{\text{\{single\}},k}$. This reflects the situation in which one performs the analysis in a single center only and one does not try to combine estimates or data.

### 4.2. Measures to quantify performance

With the BFI methodology we try to reconstruct from local inferences what we would have obtained if we had merged the data sets before doing the analysis. That means that the BFI estimators by definition cannot do better than the MAP estimators based on the combined data (the gold standard). Therefore, the parameter estimates obtained by the BFI approach are compared to those found after combining the data. Also the estimates based on a single data set and the weighted average of local estimates are compared to the combined data estimate.

Let, for a given model, $\left({\hat{\beta }}_{\text{\{BFI\}}},{\hat{\omega }}_{\text{\{BFI\}}},{\hat{M}}_{\text{\{BFI\}}}\right)$ be the BFI-estimates and $\left({\hat{\beta }}_{\text{\{com\}}},{\hat{\omega }}_{\text{\{com\}}},{\hat{M}}_{\text{\{com\}}}\right)$ the MAP estimates found after combining all data. To verify the performance of the BFI estimation method the mean squared error (MSE) is computed for each coefficient:

$${\text{MSE}}_{\beta_{k},\text{\{BFI\}}}=\frac{1}{B}\sum_{b=1}^{B}{\left({\hat{\beta }}_{\text{\{BFI\}},k}^{\left(b\right)}-{\hat{\beta }}_{\text{\{com\}},k}^{\left(b\right)}\right)}^{2},$$

where ${\hat{\beta }}_{\text{\{BFI\}},k}^{\left(b\right)}$ is the estimated value of the kth coefficient of β using the BFI method in the bth iteration, and ${\hat{\beta }}_{\text{\{com\}},k}^{\left(b\right)}$ is the estimated value of the kth coefficient using the combined data in the bth iteration. Similarly, we define the MSE for the two other estimators ${\hat{\beta }}_{\text{\{WAV\}},k}$ and ${\hat{\beta }}_{\text{\{single\}},k}$ as follows:

$$\begin{array}{rl} {\text{MSE}}_{\beta_{k},\text{\{WAV\}}} & \;=\frac{1}{B}\sum_{b=1}^{B}{\left({\hat{\beta }}_{\text{\{WAV\}},k}^{\left(b\right)}-{\hat{\beta }}_{\text{\{com\}},k}^{\left(b\right)}\right)}^{2},\\ {\text{MSE}}_{\beta_{k},\text{\{single\}}} & \;=\frac{1}{B}\sum_{b=1}^{B}{\left({\hat{\beta }}_{\text{\{single\}},k}^{\left(b\right)}-{\hat{\beta }}_{\text{\{com\}},k}^{\left(b\right)}\right)}^{2}. \end{array}$$

In all definitions of the MSE the estimate of interest is compared to the estimate that is based on the merged data. A small value means that there is hardly any loss when computing the corresponding estimator compared to what would have been obtained if all data had been merged. As a consequence of the above definitions, we do not yet measure model misspecification. A model that is misspecified can still have a small MSE, and the estimates from the true model may not necessarily have the smallest MSEs.

The square root of a diagonal element of the inverse of ${\hat{M}}_{\text{\{BFI\}}}$ is an estimate of the standard deviation of the BFI estimator of the corresponding parameter. Its accuracy (compared to what would have been found if the data sets were merged) is estimated by the MSE:

$${\text{MSE}}_{(M{)}_{\mathrm{kk}},\text{\{BFI\}}}=\frac{1}{B}\sum_{b=1}^{B}{\left({\left\{{\left({\hat{M}}_{\text{\{BFI\}}}^{\left(b\right)}\right)}^{-1}\right\}}_{\mathrm{kk}}^{1/2}-{\left\{{\left({\hat{M}}_{\text{\{com\}}}^{\left(b\right)}\right)}^{-1}\right\}}_{\mathrm{kk}}^{1/2}\right)}^{2}.$$

Since the number of baseline hazard parameters *ω* varies across the analysis models, the following MSE is employed to evaluate the efficacy of the BFI estimators of the baseline hazard functions:

$${\text{MSE}}_{\Lambda_{0},\text{\{BFI\}}}(t^{*})=\frac{1}{B}\sum_{b=1}^{B}{\left({\hat{\Lambda }}_{0,\text{\{BFI\}}}^{\left(b\right)}(t^{*})-{\hat{\Lambda }}_{0,\text{\{com\}}}^{\left(b\right)}(t^{*})\right)}^{2}.$$

To quantify the difference between the curves ${\hat{\Lambda }}_{0,\text{\{BFI\}}}(t^{*})$ and ${\hat{\Lambda }}_{0,\text{\{com\}}}(t^{*})$, we designate four distinct time points for $t^{*}$ such that the quantiles of the probability distribution of survival time of the distribution we simulate from corresponding to these points are 0.2, 0.4, 0.6, and 0.8. For the Weibull distribution with $\omega_{1}=-0.9$ and $\omega_{2}=1.8$, the time points are set to $t^{*}=(0.91,1.04,1.14,1.26)$.

In the simulation study, the mean squared errors are computed for every analysis model, for the three estimators and for multiple combinations of the sample sizes.

In [6] the asymptotic behavior of the BFI, weighted average (WAV) and single center estimators have been derived. Under the assumption of homogeneity, the BFI and the WAV estimator are asymptotically Gaussian and efficient (if the local sample sizes go to infinity and the number of centers *L* remains fixed). The single center estimator is also asymptotically Gaussian, but not efficient due to the reduced sample size. Under the assumption of heterogeneity the BFI estimator is still asymptotically efficient also for the parameters that vary across the centers. In the next section the performance of the estimators, in terms of the MSE, are compared for finite samples.

### 4.3. Simulation results

In this subsection, we present the results of our simulation study. First, for the different analysis models the MSEs of the regression parameters based on the three estimators have been computed. They are presented in the supplementary material. Consistently, across all sample sizes, the models exhibited small MSEs for the regression coefficients computed with the BFI estimates, even for small sample sizes. This holds for all models, also if the model is misspecified. This indicates that there is hardly any loss when computing the estimates with the BFI methodology, compared to the estimates that would have been found after merging the data. Also the weighted average estimator shows low MSEs. The single center estimator seems to do worse for low sample sizes, but improves if the sample size in the center increases. Further, as expected, for all models and all estimators, the MSEs tend to decrease for increasing sample sizes by decreasing variability of the estimates and possible decreasing overfitting bias. The MSEs for $\Lambda_{0}$ at four quantiles are shown in the supplementary material. It can be seen that the curves estimated by the BFI methodology are close to those estimated after combining the data sets, particularly for large local samples.

**Figure 1.**
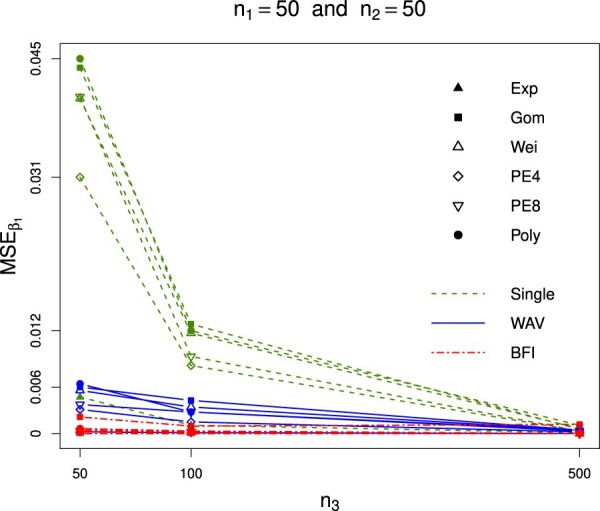
The ${\text{MSE}}_{\beta_{1},\text{\{single\}}}$, ${\text{MSE}}_{\beta_{1},\text{\{WAV\}}}$ and ${\text{MSE}}_{\beta_{1},\text{\{BFI\}}}$ for different models and as a function of the sample size in the third center $n_{3}$. The sample size in centers 1 and 2 ( $n_{1}$ and $n_{2}$) are fixed to 50. Some curves overlap. The green dashed lines (‘Single’) correspond to the ${\text{MSE}}_{\beta_{1},\text{\{single\}}}$ computed based on the estimate in the center with the highest sample size. The blue solid lines (‘WAV’) correspond to the ${\text{MSE}}_{\beta_{1},\text{\{WAV\}}}$ computed based on the weighted average of the estimates in the different centers. The red dashed lines (‘BFI’) correspond to the ${\text{MSE}}_{\beta_{1},\text{\{BFI\}}}$ and is based on the BFI estimates. In all cases the MSE is computed for comparison with the estimate for $\beta_{1}$ computed based on the merged data. The survival data were generated from a Weibull distribution.

To visualize some of our results, we plotted the MSEs for the first regression parameter across different estimators and models as a function of the sample size in the third center (Figure 1). The sample sizes in the first and second center are fixed to 50. For small sample sizes in the three centers, the gain of the BFI estimator compared to the single center becomes clearly visible. For a large sample size in one of the centers all estimators perform well; in those cases the largest data set (the third data set) simply dominates the full data set. When comparing the MSEs for the BFI and the WAV estimators, we see a similar pattern: for small sample sizes the BFI estimator outperforms the WAV estimator, but this gain disappears for an increasing total sample size. This result was expected from the theory, since under the assumption of homogeneity across centers, the BFI and WAV estimators are asymptotically efficient and, thus, follow asymptotically the same distribution. More detailed results of the simulation study are given in Table 1 in the supplementary material.

A second simulation study has been performed. In this study the life times were simulated from an exponential distribution (constant baseline hazard function). The results were very similar to the results as described above.

Increasing *γ*, the diagonal elements of the diagonal inverse covariance matrix of the prior, means stronger regularization and all parameter estimates are pushed towards zero. This leads to smaller MSE values. For *γ* close to zero, the MAP estimator is approximately equal to the maximum likelihood estimator. A similar simulation study with *γ* very small ( $10^{-9}$) yielded a similar figure as Figure 1, but with slightly larger values of the MSEs.

Better agreement between the estimates and those found after combining the data, does not necessarily mean that the estimated model is closer to the true model, as model misspecification is not measured. We therefore also computed the ${\text{MSE}}_{\beta ,\text{\{BFI\}}}$ as defined before, but replaced the estimate that is based on the merged data in the definition, by the true values of the regression parameters in the true model. This yields the values ${\text{MSE}}_{\beta ,\text{\{BFI\}},\text{\{true\}}}$ given in the supplementary material (Table 2). Now, model misspecification is also measured. The MSE values for the Weibull model (the data were simulated from a Weibull model) are smaller than those for the other analysis models. Moreover, as expected, the MSEs are much larger now. We considered two Piece-Wise Exponential models, PE4 and PE8, with, respectively, 4 and 8 equi-spaced knots. Concerning the estimation of the association parameters β (last 4 columns on the right), PE8 outperforms PE4 consistently across the various sample size scenarios considered. This suggests that the number of knots is important. A baseline hazard function with only a few knots may lead to an underfitted model. Including, naively, many equispaced knots would, in general, lead to an overfitted model, but the inclusion of a ridge regularization, as was done here, helps to contain the model complexity and thus reduce overfitting.

## 5. Application: salivary gland cancer

### 5.1. Data description

Salivary gland cancer (SGC) is a rare and diverse malignancy with over 20 subtypes. The Radboud university medical center (Radboudumc) is a tertiary SGC expertise center in the Netherlands, receiving referrals from across the country. Data was collected in a real-world database and the used data set contained data from SGC patients referred to the Radboudumc from all 8 Dutch academic medical centers and tens of smaller non-academic hospitals, with 1 to 63 patients per center [1,16]. In total, data was present for 491 patients. Of these patients, 205 have a date of death (an event). Our aim is to fit a multivariable model for the overall survival, defined as the time of diagnosis to death (of any cause), based on characteristics of the patient and the tumor. We will use the three estimators as defined before.

Based on literature and knowledge of our medical researchers, five covariates had been selected: age at diagnosis, sex, subtype, M-stage, and N-stage. The covariate ‘age’ is a continuous variable and is denoted in years, the covariate ‘sex’ is dichotomous. The variable ‘subtype’ is categorical with three levels: Salivary duct carcinoma (reference level), Adenoid cystic carcinoma, and the remainder, even more rare, subtypes combined into one category. The M-stage (metastasis stage) indicates whether the cancer has spread to other parts of the body (three categories). The covariate N-stage indicates whether the cancer is present in regional lymph nodes. In our dataset 5 categories were classified.

### 5.2. Random allocation to medical centers

**Figure 2.**
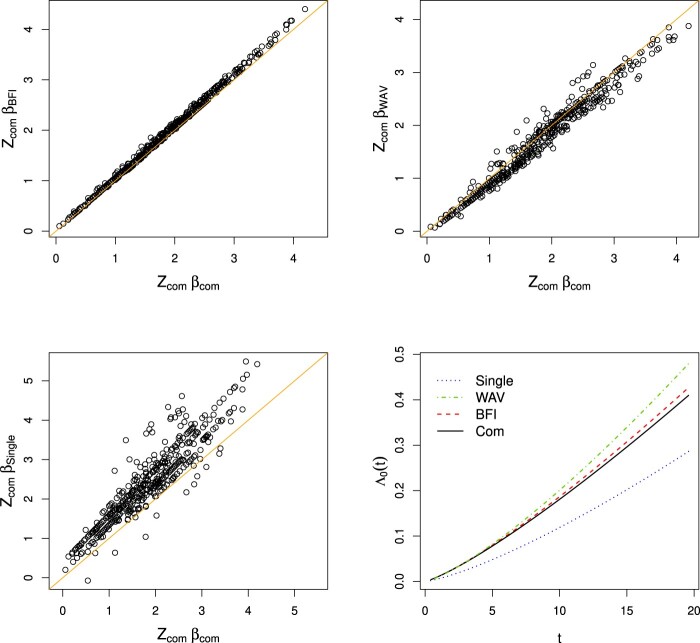
All patients have been randomly allocated to three hospitals. Scatter plots of $z^{⊤}{\hat{\beta }}_{\text{\{BFI\}}}$, $z^{⊤}{\hat{\beta }}_{\text{\{WAV\}}}$ and $z^{⊤}{\hat{\beta }}_{\text{\{single\}}}$ against $z^{⊤}{\hat{\beta }}_{\text{\{com\}}}$, respectively, for the Weibull model. Fourth plot: estimates of $\Lambda_{0}$ in the Weibull model. The priors are zero mean Gaussian distributions with diagonal inverse covariance matrices with γ=0.01 on the diagonal.

In the first data analysis we randomly distribute all patients in the data set over three fictive medical centers with, respectively, 100, 150, and 241 patients of which less than 50% had an event. The sample sizes, and thus the number of events, are relatively large in the three hospitals compared to the actual sample sizes in the centers. Still, compared to the number of model parameters, the number of events are low. In every of these three data sets (i.e. medical centers), we compute the MAP estimates of the parameters in the models described in Section 3 (Weibull, Gompertz, piecewise constant, exponentiated polynomial). For the parameter prior we took a multivariate Gaussian distribution with mean zero and a diagonal inverse covariance matrix with γ=0.01 on the diagonal. A value γ=0.01 corresponds to a variance of 100. So the MAP estimates are close to the maximum likelihood estimates. Next, for every model the MAP estimates from the three centers are combined with the BFI methodology (with the same prior for the combined data set) and the weighted average method. The single center estimator uses the data from the center with 241 patients. For all models and all patients the linear combinations $z_{\mathrm{\ell i}}^{⊤}{\hat{\beta }}_{\text{\{BFI\}}},z_{\mathrm{\ell i}}^{⊤}{\hat{\beta }}_{\text{\{WAV\}}}$ and $z_{\mathrm{\ell i}}^{⊤}{\hat{\beta }}_{\text{\{single\}}}$ are computed. Also, for every model, the MAP estimates of the model parameters were determined based on the merged data set and for every patient the corresponding linear combination $z_{\mathrm{\ell i}}^{⊤}{\hat{\beta }}_{\text{\{com\}}}$ was computed. For every model, scatter plots of the points $\left(z_{\mathrm{\ell i}}^{⊤}{\hat{\beta }}_{\text{\{BFI\}}},z_{\mathrm{\ell i}}^{⊤}{\hat{\beta }}_{\text{\{com\}}}\right)$, of the points $\left(z_{\mathrm{\ell i}}^{⊤}{\hat{\beta }}_{\text{\{WAV\}}},z_{\mathrm{\ell i}}^{⊤}{\hat{\beta }}_{\text{\{com\}}}\right)$ and of $\left(z_{\mathrm{\ell i}}^{⊤}{\hat{\beta }}_{\text{\{single\}}},z_{\mathrm{\ell i}}^{⊤}{\hat{\beta }}_{\text{\{com\}}}\right)$ were made. Since we aim to compute from the separate inference results in the local centers what would have been obtained if the data sets had been merged before the analysis, finding all points on the line *y* = *x* will correspond to a perfect fit. For the Weibull model, the three scatter plots and the estimates of $\Lambda_{0}$ are given in Figure 2. The plots for the other models are given in Supplementary Material.

From the scatter plots, we see that for all models the BFI methodology performs very well; the BFI estimates $z_{\mathrm{\ell i}}^{⊤}{\hat{\beta }}_{\text{\{BFI\}}}$ are almost exactly equal to the corresponding estimates based on the merged data. There is a minor rotation visible in the plot, possibly due to overfitting in the centers. The weighted average method also performs well for all models, but the accuracy is clearly lower than that of BFI. The performance of the single center estimator is not that good: the accuracy is low and the cloud with points seems to be rotated, possibly due to overfitting. The plots of the estimates of $\Lambda_{0}$ for the Weibull model in Figure 2 and for the other models in the supplementary material show that the BFI estimator performs well; the estimated curve is close to the estimated curve based on the combined data. The WAV estimator performs reasonably well, but the single center estimator is far from the estimated curve based on all data. We repeated the whole procedure several times and the conclusions were found to be similar.

### 5.3. Four hospitals

**Figure 3.**
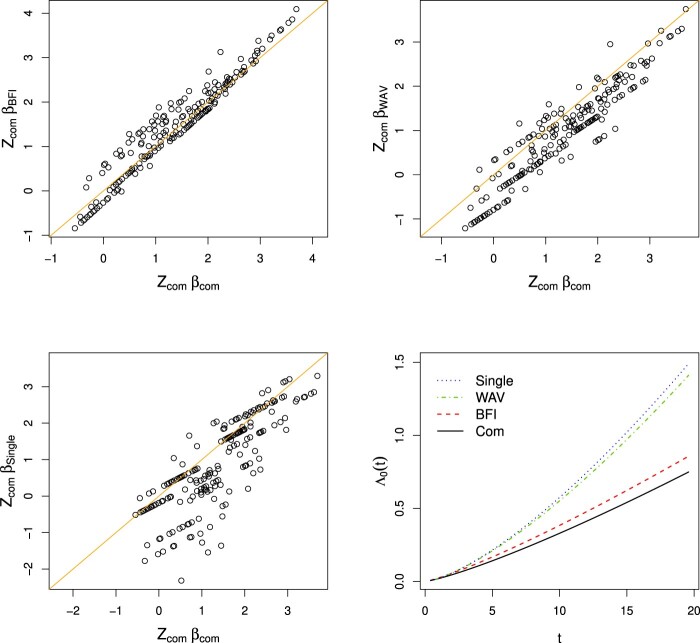
Data from four hospitals have been used. Scatter plots of $z^{⊤}{\hat{\beta }}_{\text{\{BFI\}}}$, $z^{⊤}{\hat{\beta }}_{\text{\{WAV\}}}$ and $z^{⊤}{\hat{\beta }}_{\text{\{single\}}}$ against $z^{⊤}{\hat{\beta }}_{\text{\{com\}}}$, respectively, for the Weibull model. Fourth plot: estimates of $\Lambda_{0}$ in the Weibull model. The priors equal zero mean Gaussian distributions with diagonal inverse covariance matrices with γ=0.1 on the diagonal.

We selected all centers which referred more than 30 patients each. Four centers satisfied this condition. Their sample sizes are 42, 43, 60 and 63, with, respectively, 18, 12, 39 and 28 events. We performed the BFI protocol with the Weibull, Gompertz, piecewise constant (with four intervals) and the exponentiated polynomial model (with threshold 0.10). For the prior we took a multivariate Gaussian distribution with mean zero and a diagonal inverse covariance matrix with γ=0.1 on the diagonal. A value γ=0.1 corresponds to a variance of 10. Since the number of events are really small compared to the number of model parameters, we used a larger value of *γ* to overcome overfitting. The same scatter plots as in the previous subsection were made (see Figure 3 for the Weibull model and supplementary material for the other models).

From the figures we see that the BFI estimates perform reasonably well for all models. The points are nicely scattered around the *y* = *x* line. Although for most of our models the cumulative baseline hazard function seems to be overestimated, this effect is only minor. The weighted average estimator performs less well. It seems that the linear predictor $z^{⊤}{\hat{\beta }}_{\text{\{WAV\}}}$ is underestimated; most of the points are located below the *y* = *x* line. However, in the plots with the estimates of the cumulative baseline hazard function we see that the estimate for the weighted average strategy clearly overestimates the cumulative baseline hazard function. Possibly the biases in the two estimators compensate each other. However, as our aim is to approximate the estimates that would have been found if the data had been merged, we can conclude that the weighted average estimator didn't perform well in this data example. The single center estimator is based on the data from the largest data set, so based on data of 63 patients only. Again, we compare the estimates with those that were obtained after merging the data from the four medical centers. From the plots it is clearly visible that the estimator has a poor performance. In the two smallest datasets, consisting of data from 42 and 43 patients, some variable categories had no patients. The corresponding regression parameters were estimated as zero as this is the mean of the prior distribution.

## 6. Discussion

In this paper, the BFI methodology for GLMs is extended to survival models for prediction. Survival models are more complex than GLMs by the presence of the unknown baseline hazard function. This hazard function needs to be estimated as soon as one seeks to predict survival times for new patients. Multiple parametric hazard functions of different complexity have been considered. Leaving the shape of the baseline completely unconstrained seems to be impossible without sharing patients' survival data with the central server. We considered three baseline hazard functions of fixed shape: the exponential, the Weibull and the Gompertz distribution. While these models provide a structured and interpretable approach, their low flexibility may limit their ability to capture complex hazard functions. However, especially when local data sets are small, low dimensional parametric hazard functions should be fitted to prevent overfitting. To enhance flexibility in baseline hazard modeling, we incorporated the exponentiated polynomial model, which allows the logarithm of the baseline hazard function to be represented as a polynomial of arbitrary order. The order of the polynomial function can be fixed beforehand, but can also be determined based on the data via a testing procedure. In addition, we also included the piecewise exponential model, which further improves flexibility by approximating the baseline hazard function using piecewise constant segments (if the number of intervals is not too small). The more intervals, the better the true shape of the baseline hazard can be approximated (if there is sufficient data). The positions of the knots were chosen beforehand. Usually this is not a problem as the researchers in the local centers are allowed to have contact with each other to discuss model assumptions like the location of the knots. A possible option is to use equi-spaced knots between the origin and the largest survival time recorded in all centers (this can be easily obtained before setting up the BFI procedure), as we did here. Other choices of intervals might be in principle preferred (e.g. placing the knots at the quartiles of the empirical distribution of the event times), but are difficult to implement without sharing event data. Another possibility is to adopt a Penalized Spline approach, where we use a large number of equispaced knots, but tune the (quadratic) regularization in such a way to effectively control for overfitting. In this scenario it becomes vital to have a measure of generalization error (like a cross-validation error), or a measure of goodness of fit which accounts for model complexity (e.g. AIC, BIC). These might be computed, naively, by allowing multiple communication rounds between the local centers to the central server. Whether or not these measures can actually be computed (approximately) in a BFI manner (with only one communication round) is an interesting subject for future research.

Which model is best in practice depends on the situation. If local sample sizes are large and the shape of the baseline hazard function is completely unknown, the polynomial and piecewise constant hazard functions would be preferred above the lower dimensional alternatives. However, if the local samples sizes are low, one should be careful using the piecewise exponential hazard function with many intervals and a fixed parametric shape might be preferable. Our simulations showed that while the Weibull model consistently performs well, the piecewise exponential and exponentiated polynomial models offer improved adaptability in cases where the baseline hazard deviates from standard parametric assumptions. In our real-world application on salivary gland cancer, the choice of model is particularly important given the limited sample sizes in rare cancer cohorts. The Weibull and Gompertz models provide interpretable estimates, while the piecewise exponential and exponentiated polynomial models offer more flexibility when needed.

Simulation studies have been performed to compare the performance of the BFI, WAV, and single center estimators. The results show that the BFI estimator outperforms the other estimators, but the differences in performance decrease if the local sample sizes increase. A better performance of the BFI estimator compared to the estimator based on the data of single center was expected, as the BFI estimator uses more data, and thus more information to estimate the parameters. The BFI estimators of the parameters are actually determined by a linear combination of the local estimators, but the weights are different from the weighted average estimator. Apparently this BFI combination leads to better estimators.

In the BFI framework the prior of the model parameter is a zero-mean Gaussian distribution. This assumption makes it possible to derive explicit expressions for the BFI estimator. For a large variance, the prior density is (almost) non-informative and the MAP estimates will be very similar to the maximum likelihood estimates. For parameters that are positive by definition a zero-mean Gaussian prior is not suitable. In that case a transformation of the parameter (for instance the logarithm) can be used instead, so that a Gaussian prior can be assumed for the transformed parameter. A Gaussian prior corresponds to a ridge penalty. This penalty is often added to the log likelihood to reduce overfitting of the model, especially if the sample size is small compared to the dimension of the parameter space. If one aims to do covariate selection, a lasso penalty could be used. Then, a different prior distribution must be assumed. Theoretically, this is possible in the BFI framework, but it is more complex as no explicit expression of the BFI estimator exists.

In practical applications one needs to choose the inverse covariance matrix of the prior distribution. If the matrix equals a diagonal matrix with the value *γ* on the diagonal, this *γ* coincides with the penalization parameter in a ridge regression. However, in the BFI methodology a prior is chosen for every model parameter, whereas with ridge regression only the regression parameters are penalized. The higher the value of *γ*, the stronger the penalization will be. If only one data set is available, the best value of *γ* could be selected via cross validation or other methods. In the BFI setting with multiple local centers, every center could choose its own value. The BFI strategy is constructed in such a way that the BFI estimates are approximately equal to the estimates obtained from the analysis based on the combined data set, where the sample size is higher (by definition) and less overfitting is expected to be present in the estimates. Therefore, it is not clear beforehand whether there is merit in determining the best values of *γ* in the local centers and, thus, use different values across the centers. This will be the topic of our next project.

The BFI methodology facilitates collaboration among researchers and accelerates research progress by eliminating the need for time-consuming data transfer agreements.

## Supplementary Material

Supplement.pdf

## Appendix. Generating survival data with predefined censoring rates

Here, we explain the methodology for simulating right-censored survival data from a Weibull survival distribution, censoring times from a uniform distribution, and covariate values from a Gaussian distribution. Before simulating data, we need to determine a parameter that controls the proportion of censored subjects in the final data (called the censoring rate). This is done in three steps [14]: 1) Determine the censoring probability for each subject based on subject-specific covariates; 2) Derive a censoring rate function for the study population by marginalizing covariates from the patient censoring probability function; 3) Solve the censoring parameter within the censoring rate function to achieve a specified censoring proportion. Once we have the censoring parameter, we can proceed with simulating the data.

We assume that the censoring time has density function $C∼g(c|u_{1},u_{2})=\mathrm{Uniform}(u_{1},u_{2})$ where $u_{1}$ is known and $u_{2}>u_{1}$ is an unknown parameter, and that the event time *Y* has the Weibull distribution. We let T=min(Y,C) be the observed follow-up time, *C* be independent of *Y*, and Δ=I(Y≤C) be the censoring indicator. The patient specific covariates $z_{i}$ are generated from the multivariate Gaussian distribution $z_{i}∼N(0_{p},\sigma^{2}\;I_{p})$.

By assuming the baseline hazard from $\mathrm{Weibull}(\omega_{2},1/\omega_{1})$, with $\lambda_{0}(t|,\omega_{1},\omega_{2})=\omega_{1}\omega_{2}t^{\omega_{2}-1}$, the patient censoring probability for the subject *i* can be expressed as

$$\begin{array}{rl} P(\Delta =0|\psi_{i},u_{1},u_{2},\omega_{1},\omega_{2}) & \;=P(u_{1}\leq C\leq u_{2},C\leq Y\leq \infty )\\ & \;=\int_{0}^{\infty }g(c|u_{1},u_{2})\int_{c}^{\infty }f_{Y}(t|\omega_{1},\omega_{2})\;dt\;dc\\ & \;=\frac{1}{u_{2}-u_{1}}\int_{u_{1}}^{u_{2}}\exp(-\psi_{i}\Lambda_{0}(c|\omega_{1},\omega_{2})/\omega_{1})\;dc\\ & \;=\frac{1}{u_{2}-u_{1}}\int_{u_{1}}^{u_{2}}\exp(-\psi_{i}c^{\omega_{2}})\;dc\\ & \;=\frac{\psi_{i}^{-1/\omega_{2}}}{(u_{2}-u_{1})\omega_{2}}\int_{\psi_{i}u_{1}^{\omega_{2}}}^{\psi_{i}u_{2}^{\omega_{2}}}s^{1/b-1}\exp(-s)\;ds,\;s=\psi_{i}c^{\omega_{2}},\\ & \;=\frac{\psi_{i}^{-1/\omega_{2}}}{(u_{2}-u_{1})\omega_{2}}\left[\Gamma (1/b,\psi_{i}u_{2}^{\omega_{2}})-\Gamma (1/b,\psi_{i}u_{1}^{\omega_{2}})\right], \end{array}$$

where $\psi_{i}=\omega_{1}\exp(z_{i}^{⊤}\beta )∼\mathrm{lnN}(\ln\omega_{1},\sigma^{2}\sum_{j=1}^{p}\beta_{j}^{2})$ has a lognormal distribution, and Γ(⋅,⋅) is a lower incomplete gamma function. The censoring parameter $u_{2}$ needs to be determined to yield the desirable censoring rates in simulated survival data (*π*) by numerically solving the equations below with respect to $u_{2}$:

$$\begin{array}{rl} 0=P(\Delta =0|u_{1},u_{2},\omega_{1},\omega_{2})-\pi & \;=E_{\psi_{i}}(P(\Delta =0|\psi_{i},u_{1},u_{2},\omega_{1},\omega_{2}))-\pi \\ & \;=\int_{0}^{\infty }P(\Delta =0|s,u_{1},u_{2},\omega_{1},\omega_{2})f_{\psi_{i}}(s)\;ds-\pi . \end{array}$$

By having the value of $u_{2}$, the survival data set can be simulated with predefined censoring rates *π*.
