# Supplementary material for: Bayesian federated inference for survival models
Source: J Appl Stat. 2025 Jun 4;53(2):203–23. doi: 10.1080/02664763.2025.2511932 (PMC12872092; doi:10.1080/02664763.2025.2511932)
Supplement: Supplement.pdf [file CJAS_A_2511932_SM4429.pdf]

ORIGINAL RESEARCH ARTICLE

Supplementary Material for Bayesian Federated Inference for Survival Models

Hassan Pazira, Emanuele Massa, Jetty AM Weijers, Anthony CC Coolen, Marianne A Jonker

1. Tables and Figures describing simulation studies and data analysis

In this appendix the results of the simulation studies and the data analyses are presented. In the Tables 1 and 2 the MSE for the regression parameters, their standard deviations, and the cumulative baseline hazard function in a series of time points are given, for multiple combinations of the sample sizes in three hospitals, different analysis models and the three estimators $\widehat{\beta}_{\text{BFI}}$, $\widehat{\beta}_{\text{WAV}}$ and $\widehat{\beta}_{\text{single}}$. The data were simulated from a Weibull model. In Figure 1 the performance of the BFI methodology is compared to the other proposed strategies. All salivary gland cancer patients had been randomly divided into three groups (i.e., three hospitals). In Figure 2 similar plots are shown, but this time the estimates are based on the data of the patients from the four largest medical centers.

Table 1.: Simulation results for $\text{MSE}_{\beta,\text{BFI}}$, $\text{MSE}_{\beta,\text{WAV}}$ and $\text{MSE}_{\beta,\text{single}}$ for different sets of sample sizes and survival models. The priors are zero mean Gaussian distributions with diagonal inverse covariance matrices with $\lambda = 0.01$ on the diagonal. All MSE values in this table have been multiplied by 10^3 for clarity.

		$10^3 \times \text{MSE}_{\beta,\text{BFI}}$				$10^3 \times \text{MSE}_{\beta,\text{WAV}}$				$10^3 \times \text{MSE}_{\beta,\text{single}}$			
(n_1, n_2, n_3)	model	β_1	β_2	β_3	β_4	β_1	β_2	β_3	β_4	β_1	β_2	β_3	β_4
$(50, 50, 50)$	Exp	0.00	0.01	0.01	0.01	0.20	0.21	0.23	0.22	4.41	5.11	4.25	5.24
	Gom	1.98	1.58	1.51	3.55	5.56	5.13	5.84	6.48	44.1	35.4	50.9	58.3
	Wei	0.35	0.37	0.26	0.39	5.16	4.44	5.41	4.53	40.4	32.2	30.0	39.0
	PW4	0.16	0.15	0.19	0.17	2.93	1.87	2.14	1.89	30.9	17.6	23.7	21.2
	PW8	0.30	0.23	0.22	0.49	3.55	2.96	3.06	3.54	40.6	32.2	26.2	38.6
	Poly	0.74	0.42	0.62	0.60	7.90	7.19	5.68	8.43	64.8	43.9	52.6	65.3
$(50, 50, 100)$	Exp	0.00	0.00	0.01	0.01	0.14	0.08	0.09	0.10	1.03	1.21	1.64	1.75
	Gom	0.88	0.75	0.72	1.28	4.01	2.94	3.24	3.59	13.2	11.6	14.3	11.5
	Wei	0.19	0.23	0.22	0.25	3.20	2.50	2.37	3.77	12.1	15.3	9.87	12.1
	PW4	0.07	0.08	0.08	0.10	1.37	1.17	1.23	1.10	8.20	7.80	5.35	8.96
	PW8	0.19	0.17	0.17	0.14	2.59	1.86	1.98	1.84	9.26	9.92	10.3	7.07
	Poly	0.43	0.33	0.36	0.37	5.33	2.81	1.92	5.13	20.3	11.9	9.59	14.4
$(50, 100, 100)$	Exp	0.00	0.00	0.00	0.00	0.05	0.07	0.08	0.06	1.78	1.92	1.92	1.69
	Gom	1.89	0.97	0.90	1.73	2.13	2.23	1.63	2.26	18.1	17.6	11.0	21.8
	Wei	0.14	0.08	0.09	0.07	2.23	1.31	1.21	2.33	16.5	13.1	16.4	16.4
	PW4	0.05	0.04	0.05	0.04	0.61	0.48	0.62	0.74	7.47	6.57	7.15	6.92
	PW8	0.09	0.09	0.11	0.09	1.13	0.83	0.95	1.14	13.7	8.72	10.9	10.9
	Poly	0.25	0.22	0.27	0.20	2.64	1.79	2.06	1.83	17.3	13.4	18.4	15.2
$(100, 100, 100)$	Exp	0.00	0.00	0.00	0.00	0.04	0.04	0.06	0.04	2.01	2.28	1.71	1.75
	Gom	0.63	0.31	0.33	0.63	1.08	1.35	1.18	1.39	19.6	18.2	15.8	17.9
	Wei	0.08	0.09	0.05	0.06	1.54	0.83	0.99	0.94	19.1	17.1	14.2	17.2
	PW4	0.03	0.03	0.04	0.03	0.47	0.39	0.34	0.34	10.1	7.89	7.88	8.42
	PW8	0.07	0.04	0.07	0.10	0.67	0.75	0.75	1.14	9.99	11.9	13.4	15.1
	Poly	0.12	0.11	0.10	0.11	1.33	0.99	1.30	1.47	17.2	18.4	15.3	20.1
$(50, 50, 500)$	Exp	0.00	0.00	0.00	0.00	0.02	0.02	0.01	0.02	0.08	0.10	0.09	0.12
	Gom	1.13	0.51	0.55	1.06	0.43	0.33	0.32	0.49	0.55	0.71	0.77	0.71
	Wei	0.03	0.04	0.03	0.04	0.41	0.39	0.26	0.38	0.61	0.65	0.68	0.57
	PW4	0.01	0.01	0.01	0.01	0.17	0.12	0.17	0.13	0.34	0.31	0.38	0.37
	PW8	0.03	0.02	0.02	0.03	0.25	0.17	0.17	0.19	0.59	0.39	0.48	0.52
	Poly	0.04	0.05	0.05	0.08	0.45	0.38	0.40	0.38	0.95	0.99	0.74	0.88
$(50, 100, 500)$	Exp	0.00	0.00	0.00	0.00	0.01	0.01	0.01	0.01	0.13	0.11	0.09	0.11
	Gom	0.72	0.29	0.31	0.82	0.34	0.29	0.23	0.36	0.94	1.13	1.12	1.17
	Wei	0.03	0.02	0.01	0.02	0.36	0.24	0.26	0.23	0.95	0.85	0.71	0.77
	PW4	0.01	0.01	0.01	0.01	0.07	0.12	0.08	0.10	0.58	0.58	0.42	0.41
	PW8	0.02	0.02	0.02	0.02	0.17	0.15	0.16	0.18	0.61	0.65	0.55	0.57
	Poly	0.04	0.02	0.02	0.04	0.27	0.20	0.22	0.30	1.02	0.74	0.84	0.85
$(100, 100, 500)$	Exp	0.00	0.00	0.00	0.00	0.01	0.01	0.01	0.01	0.15	0.13	0.12	0.15
	Gom	0.38	0.16	0.19	0.40	0.29	0.38	0.23	0.36	1.28	1.61	1.22	1.44
	Wei	0.01	0.01	0.02	0.02	0.25	0.26	0.15	0.25	1.13	0.87	1.15	0.99
	PW4	0.01	0.00	0.01	0.00	0.07	0.08	0.07	0.07	0.65	0.56	0.65	0.57
	PW8	0.01	0.01	0.01	0.01	0.15	0.11	0.11	0.15	0.93	0.81	0.62	0.93
	Poly	0.02	0.02	0.02	0.04	0.22	0.20	0.29	0.39	1.17	1.40	1.20	1.28
$(500, 500, 500)$	Exp	0.00	0.00	0.00	0.00	0.00	0.00	0.00	0.00	0.38	0.35	0.32	0.34
	Gom	0.03	0.01	0.01	0.03	0.08	0.05	0.06	0.08	2.78	2.25	3.02	3.77
	Wei	0.00	0.00	0.00	0.00	0.04	0.02	0.03	0.03	2.13	1.47	1.83	1.72
	PW4	0.00	0.00	0.00	0.00	0.02	0.02	0.02	0.02	1.51	1.26	1.37	1.43
	PW8	0.00	0.00	0.00	0.00	0.03	0.03	0.03	0.04	2.91	2.14	2.34	2.37
	Poly	0.01	0.00	0.00	0.00	0.06	0.05	0.05	0.05	2.76	2.28	3.07	2.90

Table 2.: Simulation results for $\text{MSE}_{\Lambda_0}(t^*)$ at four different quantiles, $\text{MSE}_{(\mathbf{M})_{kk},\text{BFI}}$ and $\text{MSE}_{\boldsymbol{\beta},\text{BFI,true}}$ of the regression coefficients for different sets of sample sizes and survival models. The priors are zero mean Gaussian distributions with diagonal inverse covariance matrices with $\lambda = 0.01$ on the diagonal. For clarity, all MSE values are scaled by 10^3, except $\text{MSE}_{(\mathbf{M})_{kk},\text{BFI}}$, scaled by 10^6. Bold numbers indicate the best model, as the data were generated from it.

		$10^3 \times \text{MSE}_{\Lambda_0}(t^*)$				$10^6 \times \text{MSE}_{(\mathbf{M})_{kk},\text{BFI}}$				$10^3 \times \text{MSE}_{\boldsymbol{\beta},\text{BFI,true}}$			
(n_1, n_2, n_3)	model	20%	40%	60%	80%	β_1	β_2	β_3	β_4	β_1	β_2	β_3	β_4
$(50, 50, 50)$	Exp	0.04	0.06	0.07	0.08	0.98	1.12	1.01	1.10	230	106	105	226
	Gom	0.54	2.59	12.2	70.7	8.55	15.8	15.3	12.5	25.5	25.5	19.4	29.5
	**Wei**	**0.23**	**1.23**	**4.35**	**15.6**	**12.5**	**12.9**	**11.7**	**7.85**	**17.0**	**14.2**	**10.8**	**15.2**
	PW4	0.66	1.56	3.01	8.17	7.97	5.55	7.23	6.49	26.1	18.3	15.2	33.6
	PW8	1.49	3.72	8.57	23.8	8.19	9.20	6.15	7.22	18.0	11.6	14.1	14.3
	Poly	0.75	2.51	6.45	17.4	14.8	8.74	14.2	10.3	15.9	12.7	19.0	20.1
$(50, 50, 100)$	Exp	0.03	0.04	0.04	0.05	0.40	0.35	0.38	0.50	224	103	108	228
	Gom	0.24	1.01	4.35	23.8	5.40	5.68	5.84	6.35	22.3	19.0	16.8	19.3
	**Wei**	**0.17**	**0.85**	**2.90**	**10.1**	**4.30**	**4.94**	**3.62**	**4.30**	**7.43**	**8.81**	**10.1**	**11.9**
	PW4	0.33	0.80	1.50	3.64	2.89	3.20	2.90	2.39	26.9	14.1	13.6	26.8
	PW8	0.81	2.19	4.60	10.2	3.64	3.42	3.83	2.72	16.4	13.2	11.2	12.0
	Poly	0.37	1.26	3.31	9.09	5.28	5.20	4.60	4.98	16.8	15.3	13.3	17.5
$(50, 100, 100)$	Exp	0.02	0.02	0.03	0.03	0.27	0.23	0.27	0.20	227	98.2	102	228
	Gom	0.23	0.71	3.41	22.4	3.37	2.94	4.58	2.75	18.0	11.0	12.3	17.2
	**Wei**	**0.07**	**0.40**	**1.44**	**5.24**	**2.17**	**2.41**	**2.93**	**2.27**	**8.52**	**5.59**	**8.46**	**8.05**
	PW4	0.18	0.46	0.83	1.90	1.36	1.45	1.47	1.21	25.5	15.3	13.8	23.4
	PW8	0.43	1.10	2.72	6.59	2.55	1.81	1.83	1.89	11.4	7.64	9.03	12.1
	Poly	0.20	0.71	1.93	5.57	2.16	2.59	3.94	2.96	8.28	8.91	8.20	8.24
$(100, 100, 100)$	Exp	0.01	0.01	0.02	0.02	0.17	0.15	0.10	0.10	232	100	101	227
	Gom	0.12	0.44	1.96	11.7	2.33	1.59	2.13	2.45	13.4	8.80	8.36	16.5
	**Wei**	**0.06**	**0.30**	**1.03**	**3.44**	**1.63**	**1.53**	**1.63**	**1.80**	**6.93**	**6.10**	**6.23**	**7.78**
	PW4	0.15	0.35	0.61	1.32	0.74	0.98	0.61	0.89	20.8	11.7	13.2	21.0
	PW8	0.29	0.74	1.60	4.19	0.94	1.39	1.00	1.62	6.60	6.19	6.66	9.59
	Poly	0.11	0.42	1.18	3.54	1.85	2.01	1.57	1.91	12.6	9.14	7.85	8.87
$(50, 50, 500)$	Exp	0.00	0.00	0.01	0.01	0.02	0.01	0.02	0.02	226	100	100	228
	Gom	0.09	0.06	0.45	7.10	0.17	0.21	0.24	0.22	10.7	6.73	7.77	12.0
	**Wei**	**0.02**	**0.09**	**0.30**	**0.98**	**0.13**	**0.22**	**0.16**	**0.15**	**3.31**	**3.35**	**2.40**	**2.78**
	PW4	0.04	0.10	0.18	0.40	0.10	0.11	0.12	0.11	25.8	11.3	12.2	26.3
	PW8	0.09	0.21	0.45	1.04	0.13	0.12	0.13	0.14	9.67	6.88	6.39	11.6
	Poly	0.04	0.12	0.33	0.94	0.29	0.28	0.23	0.42	6.34	4.27	3.23	6.21
$(50, 100, 500)$	Exp	0.00	0.00	0.00	0.00	0.01	0.01	0.02	0.02	227	101	101	226
	Gom	0.07	0.09	0.41	4.84	0.19	0.24	0.15	0.31	8.71	5.43	4.47	9.21
	**Wei**	**0.01**	**0.06**	**0.21**	**0.72**	**0.20**	**0.14**	**0.10**	**0.13**	**2.92**	**2.26**	**2.95**	**2.70**
	PW4	0.03	0.08	0.15	0.31	0.07	0.07	0.08	0.08	21.5	10.3	10.9	22.0
	PW8	0.07	0.18	0.38	0.92	0.09	0.11	0.11	0.10	9.26	4.99	4.67	9.60
	Poly	0.03	0.10	0.28	0.79	0.15	0.18	0.16	0.17	6.19	4.47	4.65	7.18
$(100, 100, 500)$	Exp	0.00	0.00	0.00	0.00	0.01	0.01	0.01	0.01	227	100	100	226
	Gom	0.04	0.05	0.27	2.68	0.19	0.22	0.20	0.21	8.38	4.71	5.62	7.79
	**Wei**	**0.01**	**0.05**	**0.19**	**0.68**	**0.12**	**0.10**	**0.15**	**0.15**	**2.54**	**2.15**	**2.51**	**2.81**
	PW4	0.02	0.06	0.10	0.22	0.07	0.05	0.07	0.08	19.5	9.17	10.3	21.0
	PW8	0.05	0.13	0.30	0.68	0.11	0.09	0.07	0.09	6.00	3.10	3.70	6.22
	Poly	0.02	0.08	0.23	0.71	0.15	0.21	0.17	0.28	5.91	3.66	4.46	5.36
$(500, 500, 500)$	Exp	0.00	0.00	0.00	0.00	0.00	0.00	0.00	0.00	227	101	101	227
	Gom	0.00	0.02	0.08	0.44	0.05	0.03	0.03	0.03	5.36	2.83	2.24	4.83
	**Wei**	**0.00**	**0.01**	**0.03**	**0.09**	**0.01**	**0.01**	**0.01**	**0.01**	**1.51**	**0.98**	**1.22**	**1.07**
	PW4	0.01	0.01	0.03	0.05	0.01	0.01	0.01	0.01	17.4	8.50	8.29	17.3
	PW8	0.01	0.03	0.06	0.15	0.01	0.01	0.01	0.01	3.21	2.17	1.81	3.59
	Poly	0.00	0.02	0.05	0.16	0.03	0.02	0.04	0.03	3.21	2.39	2.78	4.33

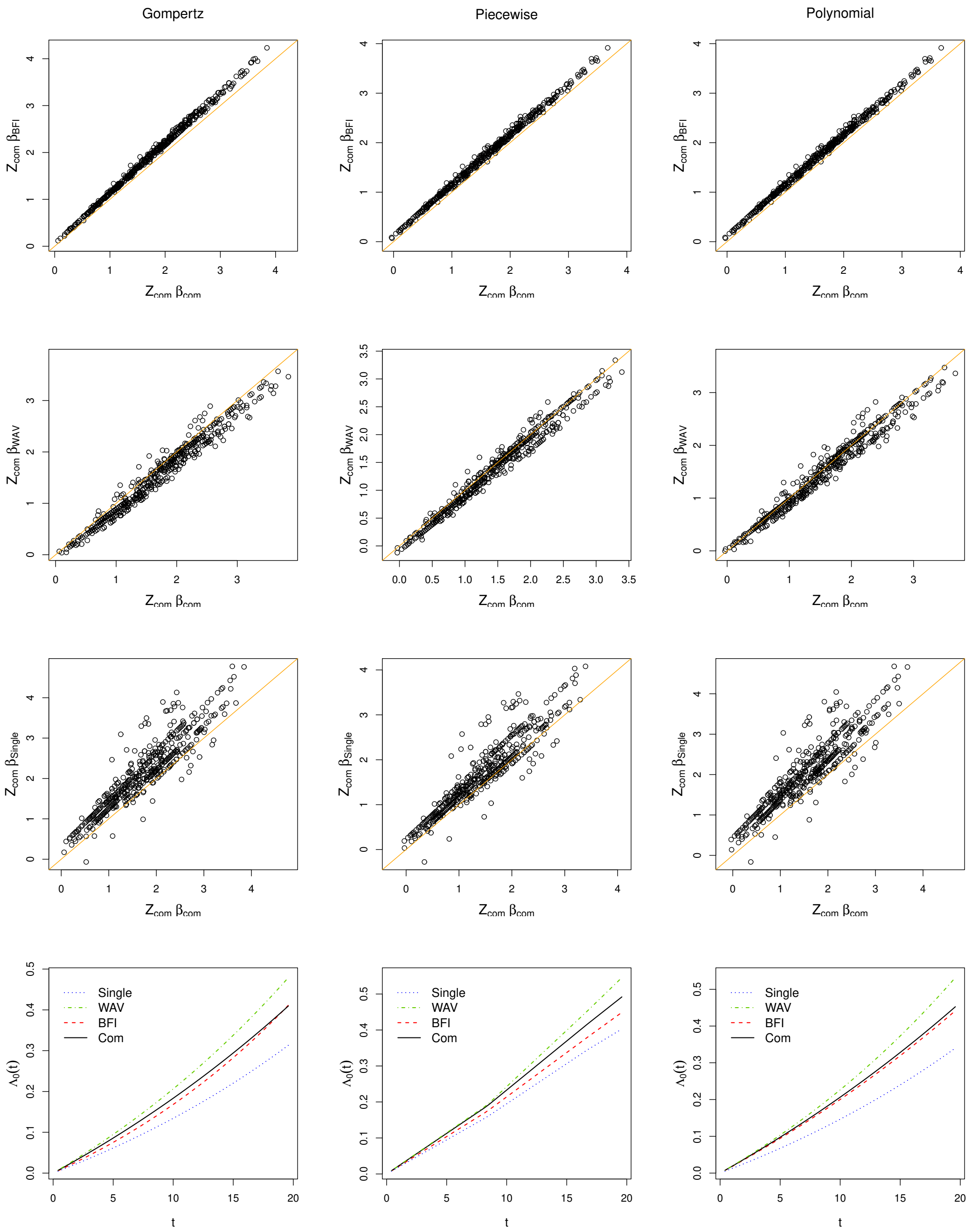

Figure 1.: All patients were randomly allocated to three medical centers. Scatter plots of $\boldsymbol{z}^\top\widehat{\boldsymbol{\beta}}_{\text{BFI}}$, $\boldsymbol{z}^\top\widehat{\boldsymbol{\beta}}_{\text{WAV}}$ and $\boldsymbol{z}^\top\widehat{\boldsymbol{\beta}}_{\text{single}}$ against $\boldsymbol{z}^\top\widehat{\boldsymbol{\beta}}_{\text{com}}$ in the first, second and third row, respectively, for three models: Gompertz (first column), piecewise constant with four intervals (second column), exponentiated polynomial (third column). Fourth row: estimates of Λ_0 in the three models: Gompertz (first plot), piecewise constant (second plot), exponentiated polynomial (third plot). The priors equal zero mean Gaussian distributions with diagonal inverse covariance matrices with $\gamma = 0.01$ on the diagonal.

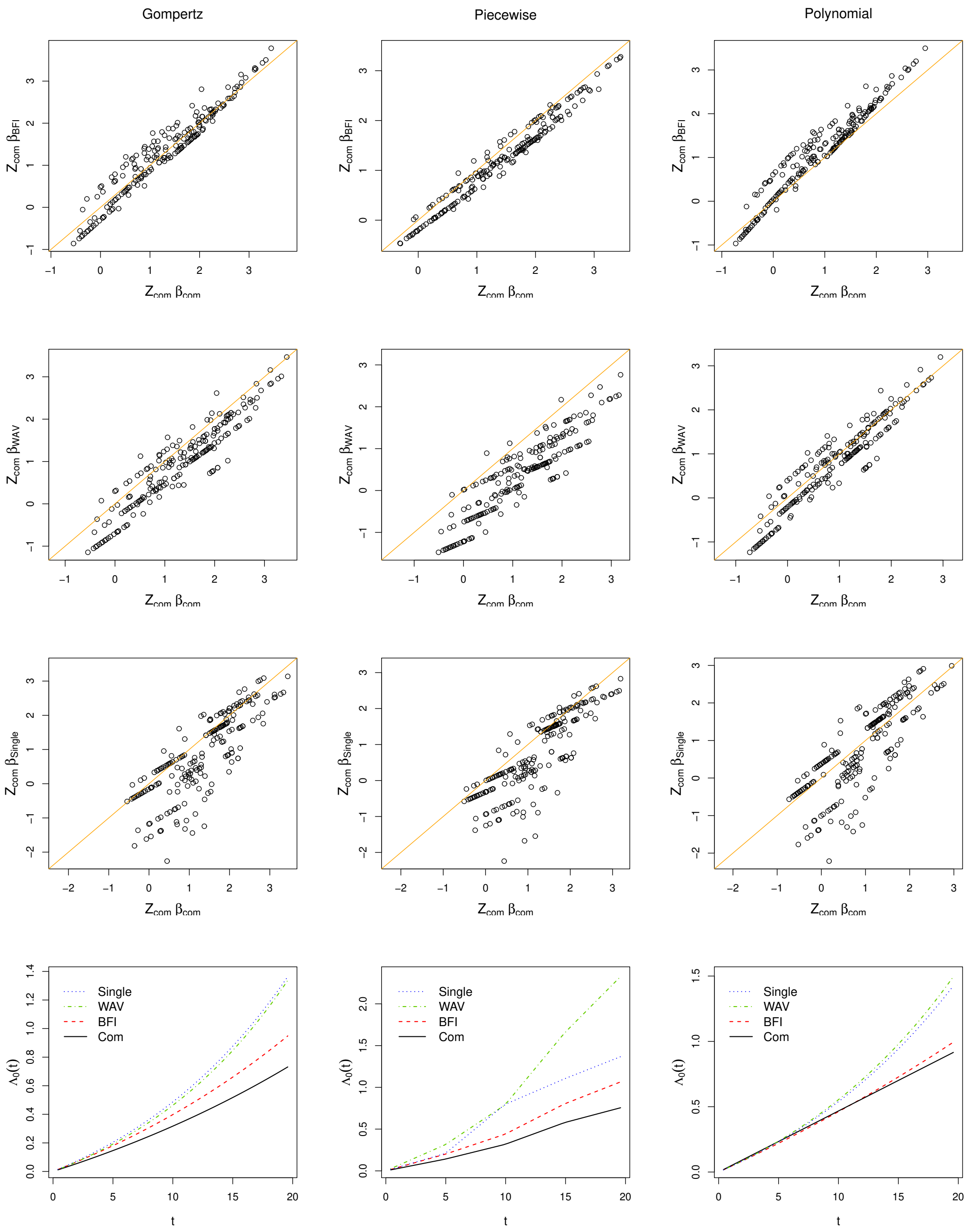

Figure 2.: Data from four hospitals. Scatter plots of $z^\top\widehat{\beta}_{\text{BFI}}$, $z^\top\widehat{\beta}_{\text{WAV}}$ and $z^\top\widehat{\beta}_{\text{single}}$ against $z^\top\widehat{\beta}_{\text{com}}$ in the first, second and third row, respectively, for 3 models: Gompertz (first column), piecewise constant with four intervals (second column), exponentiated polynomial (third column). Fourth row: estimates of Λ_0 in the three models: Gompertz (first plot), piecewise constant (second plot), exponentiated polynomial (third plot). The priors equal zero mean Gaussian distributions with diagonal inverse covariance matrices with $\gamma = 0.1$ on the diagonal.
